# Harnessing halophyte‐derived allelochemicals and signaling molecules to enhance salinity tolerance in crops

**DOI:** 10.1002/ajb2.70076

**Published:** 2025-07-21

**Authors:** Gyöngyi Székely, Csengele Barta

**Affiliations:** ^1^ Hungarian Department of Biology and Ecology Faculty of Biology and Geology, Babeș‐Bolyai University 5‐7 Clinicilor St. Cluj‐Napoca 400006 Romania; ^2^ Institute for Research‐Development‐Innovation in Applied Natural Sciences Babeș‐Bolyai University 30 Fântânele St. Cluj‐Napoca 400294 Romania; ^3^ Centre for Systems Biology, Biodiversity and Bioresources (3B) Babeș‐Bolyai University 5‐7 Clinicilor St. Cluj‐Napoca 400006 Romania; ^4^ Department of Biology Missouri Western State University 4525 Downs Drive, Agenstein‐Remington Halls, St. Joseph 64507 USA

**Keywords:** abiotic stress mitigation, allelopathy and allelochemicals, biostimulants, phytohormones, plant‐plant interactions, plant secondary metabolites, salinity adaptation, salt stress tolerance, signal molecules, sustainable agriculture

## Abstract

**Premise:**

Soil salinization is a growing global challenge that significantly reduces agricultural productivity by impairing seed germination, growth, and yield. While conventional crops have limited tolerance to high salinity, halophytes are promising biological models for developing strategies to sustain agriculture in saline environments and support global food security. This review addresses the potential of halophyte‐produced allelochemicals and related signaling molecules to mitigate the impacts of salinization, a topic of growing relevance for sustainable agriculture in a changing climate.

**Methods:**

We surveyed and synthesized current research on halophyte allelochemicals and complementary plant‐derived molecules and discussed their roles in enhancing resilience to salt stress. Emphasis was placed on distinguishing true allelochemicals from other biologically active compounds and evaluating their applications in plant stress management.

**Results:**

Conventional allelochemicals that are synthesized and released into the environment by halophytes modulate plant responses and may enhance their salt stress resistance. In addition, phytohormones, polyamines, and microbial metabolites have also demonstrated significant hardening effects by enhancing plant tolerance to salinity. Halophytes also provide additional ecosystem benefits as biofuel, forage, or edible crop sources and play a role in phytoremediation.

**Conclusions:**

Using halophyte‐derived allelochemicals and complementary signaling molecules offers a viable, environmentally friendly way to increase crop production in saline areas, reduce soil salinization, and conserve freshwater. Future research is expected to focus on optimizing application strategies, evaluating environmental risks, and integrating allelopathy‐based approaches into sustainable agricultural systems to enhance crop resilience in the face of climate change.

The United Nations (UN) estimates the world's population to rise to 10.2 billion by 2050, increasing food and resource demands (Muhammad et al., 2019). Concurrently with inadequate precipitation, extended heat events, inappropriate agricultural practices, and irrigation with saline water, all of which contribute to the large‐scale expansion of saline areas unsuitable for crop cultivation, the negative effects of climate change are also intensifying. With almost half of arable land projected to be salt‐affected by 2050, salinized soils are expected to expand by 10% each year (Shrivastava and Kumar, 2015). The distribution of salt‐affected areas worldwide shows a concerning trend. Figure 1 highlights the breadth of soil salinization on agricultural lands (FAO, 2021).

**Figure 1:**
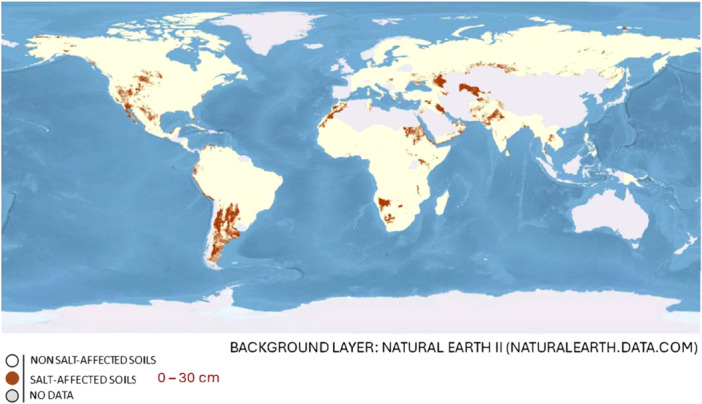
Global distribution of salinity‐affected regions, based on soil salinity measurements at 0–30 cm depth. *Source*: Food and Agriculture Organization (FAO, 2021).

Worldwide, 85% of the land is slightly to moderately affected by salinization, while the remaining areas pose severe restrictions on crop cultivation (Wicke et al., 2011). Reflecting the amount of dissolved salts in soil water, which directly affects plant water absorption capacities, soil salinity is quantified through soil electrical conductivity (EC), expressed in deciSiemens per m (dS/m) units. Soil EC also serves as a proxy for soil nutrient status, structure, fertility, and health.

Soils with EC values exceeding 4 dS/m at 20°C and with exchangeable Na^+^ content above 15% are deemed saline (Shrivastava and Kumar, 2015). Slightly saline soils (2–4 dS/m) can reduce yield in sensitive crops, while moderate (4–8 dS/m) and severe salinity (>8 dS/m) cause pronounced, often severe physiological and metabolic impairments, accompanied by substantial yield losses (Rengasamy, 2010; FAO, 2021). Salinization can also reduce soil permeability, alter microbial community structure and composition, limit nutrient availability, and amplify the negative impacts of drought (Rengasamy, 2010).

A unique example of a moderately saline ecosystem is that of Cojocna, Romania (Figures 2 and 3), where soil electrical conductivity (EC) values range between 3.14 and 4.45 dS/m, corresponding to 24.35–41.35 mM NaCl concentrations (Brezestean et al., 2018). These soils support halophytic plant species such as petrosimonia (*Petrosimonia triandra)*, a notable species equipped with a variety of salinity tolerance mechanisms (Schaminée et al., 2018; Podar et al., 2019; Székely et al., 2022). In Romania, *P. triandra* is characteristic of halophytic communities (Podar et al., 2019; Dité et al., 2021; Székely et al., 2022) and protected as part of Natura 2000 site ROSCI0238, Suatu–Cojocna–Crairât (European Environment Agency, 2025) (Figure 3). The rhizosphere of *P. triandra* has been identified as a promising niche for isolating salt‐tolerant plant‐growth‐promoting rhizobacteria (PGPR) to develop microbial applications for enhancing crop resilience (Székely et al., 2022). Aside from its characteristic flora, Cojocna's hypersaline lakes, formed over Miocene salt deposits, contribute to its distinctive limnological and microbial diversity (Brezestean et al., 2018). These features underscore the importance of conserving Cojocna's saline landscapes, which serve as natural field laboratories for studying plant and ecosystem adaptations to salinity.

**Figure 2:**
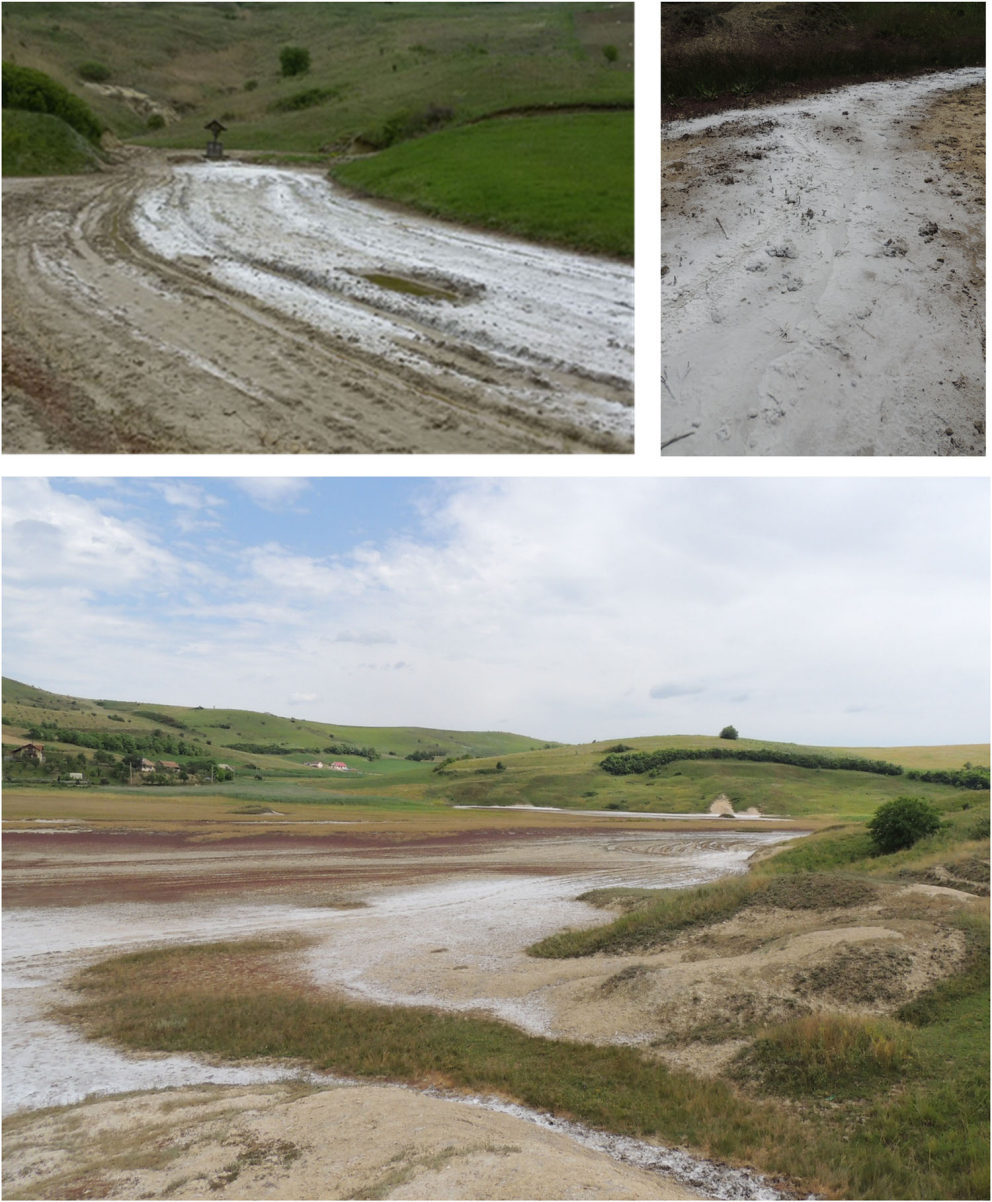
Representative photographs of salinized land in Cojocna, Romania (46.7495°N, 23.8367°E). This site is protected under the Natura 2000 network as ROSCI0238 Suatu–Cojocna–Crairât, designated under the Nature and Habitats Directive of the European Environment Agency (European Environment Agency, 2025). Photographs: G. Székely.

**Figure 3:**
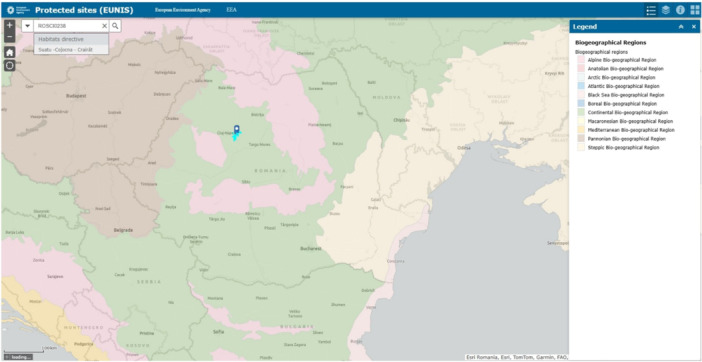
Map of Natura 2000 protected area ROSCI0238 Suatu–Cojocna–Crairât (Cojocna, Romania; coordinates: 46.7495° N, 23.8367° E), generated using the European Environment Agency's EUNIS Protected Site Map via the EEA/ESRI GIS Interactive Mapping Service. Basemap colors indicate distinct biogeographical regions. The turquoise area and site marker denote the ROSCI0238 Suatu–Cojocna–Crairât site (European Environment Agency, 2025).

Increasing soil salinization compromises crop production, long‐term food security, and sustainability. In water‐limited areas, conventional methods to reduce these effects—using chemical amendments or breeding salt‐tolerant crops—have had little success. Thus, exploiting halophyte adaptations and plant‐derived signaling molecules is attracting interest as a sustainable alternative for minimizing the impacts of salinity stress on crops.

We focused on two fundamental questions for this review: (1) How do halophytes contribute to mitigating the impacts of soil salinization on plants? (2) How can the allelopathic potential of halophytes be harnessed to enhance the salt tolerance of sensitive food crops, with the goal to translate halophyte adaptations into knowledge to support sustainable practices in agriculture (Rozema and Flowers, 2008; Shabala, 2013; Parihar et al., 2015).

## SALT STRESS

### Halophyte adaptations to saline environments

Saline soils (EC > 4 dS/m) pose challenging conditions for crop growth and development; biomass, yield, and grain or fruit quality are already being reduced by moderate soil electrical conductivities (ECs) (Shahbaz and Ashraf, 2013; Zörb et al., 2018). In 2021, the Food and Agriculture Organization (FAO) of the United Nations estimated that annual crop yield losses attributable to soil salinization reached US$31 million, with up to 46 million hectares of land losing their production potential each year (FAO, 2021).

Halophytes, a group with high species diversity, which represent about 2% of terrestrial plants (Glenn et al., 1999), can tolerate saline soils and complete their life cycle in soils exceeding 200 mM NaCl (Flowers and Colmer, 2015). Salt‐tolerant species such as common seepweed (*Suaeda salsa*; Guo et al., 2015), herbaceous seepweed (*Suaeda maritima*) (Flowers and Colmer, 2015) and old man saltbush (*Atriplex numularia*; de Araújo et al. 2006) have improved seed vigor and enhanced growth in soils with up to 300 mM NaCl, while common glasswort (*Salicornia europaea*), fireweed (*Kochia scoparia*) and raghal (*Atriplex leucoclada*) thrive in extreme saline conditions, at over 500 mM NaCl in the soil (Mohammadi and Kardan, 2015). Species like dwarf glasswort (*Salicornia bigelovii*) and other saltbush species (*Atriplex* spp.), which grow and reproduce in extreme salinity, are of particular interest for their potential to serve as models for improving salt tolerance in conventional crops.

In sensitive species, the salt‐stress‐induced reduction in seed germination capacity results from decreased osmotic potential around the seed, which inhibits water uptake (Welbaum et al., 1990). Elevated Na^+^ and Cl^–^ concentrations (421 mM NaCl ~ EC: 40 dS/m) present in hypersaline soils can also be toxic to seeds (Khajeh‐Hosseini et al., 2003). Overall, salinity negatively affects photosynthesis; lipid metabolism; DNA, RNA, and protein synthesis; and mitosis in sensitive plant species (Niu et al., 2013).

While increasing soil salinity constrains seed germination and inhibits plant growth, physiological performance, and yield in sensitive species (Abbas et al., 2021; Liu et al., 2021; Yaghoubian et al., 2022), the ability of halophytes to thrive in environments with salinity levels similar to, or exceeding seawater salt concentrations is attributed to adaptations that counteract the toxic effects of high rhizospheric salt concentrations. These include specialized, regulated ion transport systems, salt exclusion from roots or at the leaf level, and the sequestration of toxic ions into vacuoles (Shabala and Mackay, 2011; Flowers et al., 2015).

Tolerance mechanisms also include morphological, physiological and biochemical adaptations, osmotic adjustment, altered nutrient ratios (in particular K^+^/Na^+^ ratios), enhanced production of antioxidant enzymes, limited evapotranspiration achieved by the reduction of leaf areas, variations in photosynthetic pigments and the recruitment of PGPR (Shrivastava and Kumar, 2015; Podar et al., 2019; Sarwar et al., 2019; Székely et al., 2022). Many halophytic species evolved salt glands that excrete salts onto the leaf surface or sequester salt into the central vacuole to avoid cytotoxicity (Flowers and Colmer, 2015; Santos et al., 2015; Kuster et al., 2020). Halophytes also have an efficient scavenging system that includes enzymes and non‐enzymatic compounds to detoxify the high levels of reactive oxygen species (ROS) that are generated in response to stress. Cellular osmotic balance is modulated through the accumulation of compatible solutes with cell‐protecting osmotic activity, also called osmolytes. These cytoplasmic molecules include proline, glycine betaine, β‐alanine, sugars, and sugar alcohols as sorbitol, or choline derivatives as the choline‐*O*‐phosphate. Halophytes often accumulate only one dominant solute (Tipirdamaz et al., 2006; Székely et al., 2008; Lugan et al., 2010; Slama et al., 2015). For example, common glasswort (*Salicornia europaea*) primarily accumulates glycine betaine, and herbaceous seepweed (*Suaeda maritima*) accumulates proline. Other species, however, may simultaneously accumulate multiple compatible solutes, combining their osmoprotectant effects to maximize stress resilience (Gagneul et al., 2007). For example, Mediterranean saltbush (*Atriplex halimus*) stores glycine betaine and choline‐*O*‐sulfate, the sea lavender (*Limonium latifolium*) accumulates sugars and sugar alcohols such as sucrose and sorbitol, and common ice plant (*Mesembryanthemum crystallinum*) produces high levels of proline and sugar to maintain osmotic balance in saline conditions (Tipirdamaz et al., 2006; Székely et al., 2008; Lugan et al., 2010; Slama et al., 2015). In addition, exogenous proline also improves antioxidant capacity, enhances photosynthesis, and induces increments in phenolic contents to strengthen the defense system of celery (*Apium graveolens*) plants against salt stress (Gao et al., 2023). Apart from osmotic control, compatible solutes also help to stabilize proteins and membranes, preserving cellular redox balance and safeguarding enzyme activity under ionic and osmotic pressures (Szabados and Savouré, 2010). Glycine betaine accumulation, for instance, increases salinity resilience and photosynthetic efficiency in halophytes such as Mediterranean saltbush and seepweed and in engineered glycophytes—salt‐sensitive plants adapted to low‐salinity environments—such as transgenic thale cress (*Arabidopsis thaliana*) and rice (*Oryza sativa*) (Ashraf and Foolad, 2007).

### Halophytes in agriculture

Owing to their unique ability to grow, develop in, and benefit from saline environments, halophytes support multiple applications in agriculture and are valuable biological resources for developing novel strategies to mitigate the impacts of salinization and climate change. Some of their key applications are briefly discussed next.

#### Phytoremediation

Halophytes can act as bio‐desalinating agents to effectively eliminate excess salt from soils. By accumulating salt in their tissues, they help to restore soil fertility for crop cultivation, which could be crucial for increasing land area for agriculture (Hasanuzzaman et al., 2014; Karakas et al., 2020). Halophytes such as the common glasswort, saltbush (*Atriplex verrucifera*), saltwort (*Salsola crassa*), and jointed glasswort (*Halocnemum strobilaceum*) could be used as efficient desalinating species to restore arid and semiarid regions (Ahmadi et al., 2022).

#### Pasture cover crops and livestock feed

Where high salinity suppresses conventional forage crops, halophyte‐based pastures offer sustainable sources of feed for cattle. Dwarf glasswort (*Salicornia bigelovii*), saltbush (*Atriplex* spp.), and seepweed (*Suaeda* spp.) are high‐value fodder, forage, and grazing crops that can fill seasonal food gaps (Al‐Azzawi and Flowers, 2022; Hasnain et al., 2023).

#### Salty vegetable crops

Glasswort (*Salicornia salsola*), saltbush (*Atriplex* spp.), sea rocket (*Cakile* spp.), seepweed (*Suaeda* spp.), and plantain (*Plantago* spp.) are used as salty vegetables in culinary applications (Tug and Yaprak, 2017; Lombardi et al., 2022). Due to their unique flavors and high nutrient content, these halophytes cater to specialized markets and promote crop diversity.

#### Biofuel crops

Many halophytic species produce large amounts of biomass and lipids, ideal qualities for biofuel generation. For instance, glasswort species have been extensively studied for their potential use in biodiesel production in saline environments (AlYammahi et al., 2024).

#### Ornamental plants

The aesthetic appeal of halophytes and their ability to thrive in harsh environments make them a creative solution for the economic utilization of saline soils, while also conserving biodiversity (Ventura et al., 2014). Over 1198 plant species are listed in the HALOPH database as halophytic ornamentals (Santos et al., 2015; eHALOPH, 2025).

#### Medicinal plants

Halophytes produce bioactive molecules with antiviral, antifungal, and antibacterial properties, which have been used or have potential to treat human infectious and non‐infectious diseases (Ferreira et al., 2022). For example, common glasswort (*Salicornia herbacea*) is effective in treating diabetes, oxidative stress, inflammation, hepatitis, asthma, and cancer (Essaidi et al., 2013).

#### Saline agriculture and freshwater conservation

As already mentioned, halophytes can be grown in soils with elevated salinity where traditional crops cannot grow. Glasswort species can be irrigated with saline water or even seawater, so that freshwater can be used to irrigate conventional crops (Puccinelli et al., 2024). This practice helps alleviate freshwater overuse for irrigation, contributing to sustainable agriculture and food security.

## ALLELOPATHY AND ITS ROLE IN ENHANCING SALT STRESS TOLERANCE FOR SUSTAINABLE AGRICULTURE

### Conventional allelopathic metabolites

Many biotechnological and molecular engineering studies have focused on enhancing crop productivity by increasing plant tolerance to salt (Xu et al., 2014; Székely and Barta, 2022; Tarolli et al., 2024). An efficient and environment‐friendly alternative could also be achieved by harnessing the effects of natural, plant‐produced allelochemicals (Weston and Duke, 2003; Kong et al., 2019).

Allelopathy is a plant–plant chemical interaction in which one plant influences either positively or negatively the development, physiology, behavior, and survival of its neighbors, through the synthesis and release of allelochemicals into their immediate proximity (Farooq et al., 2011a; Mushtaq et al., 2020). A wide range of secondary plant metabolites serve as allelochemicals that modulate plant–plant and plant–environment interactions and confer a competitive advantage. Some of these allelochemicals are also highly beneficial in mitigating abiotic stress, such as the impacts of salinity.

Conventional allelopathic metabolites are phenolic, terpenoid, alkaloid, flavonoid, and benzoxazinoid metabolites (Weston and Duke, 2003; Kong et al., 2019). Their structure and properties determine their modes of action, mobility in plants and the environment, environmental persistence, and interactions with plant metabolic pathways.

Phenolics, the most abundant and well‐studied allelochemicals, include simple phenolic acids (e.g., ferulic, *p*‐coumaric, and vanillic acids) and more complicated structures, such as flavonoids (e.g., catechin and quercetin) and tannins. In the recipient plants, phenolic allelochemicals interfere with cell wall synthesis, disrupt membrane integrity, limit nutrient absorption, inhibit root elongation, and cause oxidative stress (Li et al., 2010; Cheng et al., 2024). Sorgoleone, exuded by the roots of sorghum species (*Sorghum* spp.), is a potent inhibitor of photosystem II (Dayan et al., 2010). In addition to their inhibitory effects, many phenolics also contribute to salt stress mitigation by modulating antioxidant defenses and osmotic balance at low concentrations (Kong et al., 2019).

Terpenoids and alkaloids are often emitted as volatiles. Volatile monoterpenes and sesquiterpenes such as cineole and camphor can prevent seed germination and root elongation in nearby plants (Inderjit and Duke, 2003). Alkaloids such as nicotine synthesized by tobacco species (*Nicotiana* spp.) or gramine, produced mainly by grasses, can disrupt signal transduction pathways, enzymatic activities, and energy metabolism in other species (Weston and Duke, 2003).

The benzoxazinoids DIBOA (2,4‐dihydroxy‐1,4‐benzoxazin‐3‐one) and DIMBOA (2,4‐dihydroxy‐7‐methoxy‐1,4‐benzoxazin‐3‐one), synthesized by maize (*Zea mays*) and other grass species, have been investigated for their roles in defense against herbivores and microbial pathogens and for their potent allelopathic effects (Niemeyer, 2009; Kong et al., 2019).

Allelopathic interactions are efficient mechanisms that allow a particular plant species to outcompete its neighbors and to invade native plant communities through two primary strategies. One involves establishing a virtual monoculture in a diverse plant community. However, such monocultures are relatively rare and are often considered anomalies. The more common and high‐impact strategy is allelopathic suppression of neighboring species that are naïve to the specific allelochemical(s) and thus highly vulnerable (Hierro and Callaway, 2003).

One of the most thoroughly studied examples of allelopathy‐supported invasions involves knapweed species (e.g., diffuse knapweed [*Centaurea diffusa*]) of Eurasian origin. Hierro and Callaway (2003) documented that Eurasian neighbors have adapted to the allelochemicals [e.g., (±)‐catechin and 8‐hydroxyquinoline] produced by diffuse knapweed, whereas its North American neighbors—encountering these bioactive molecules for the first time—have not; thus, germination and growth of the naïve species are significantly more suppressed. Similar in structure to secondary plant metabolites involved in plant defense responses, the flavonoid‐derived flavan‐3‐ol, (±)‐catechin, released into the rhizosphere by both the diffuse and the spotted knapweed (*Centaurea maculosa*), accumulates in the root zone of neighboring plants such as Idaho fescue (*Festuca idahoensis*) and prairie Junegrass (*Koeleria macrantha*) and suppresses their germination and growth (Bais et al., 2003). Catechins enhance ROS production in the root tissues of sensitive species, causing oxidative damage, membrane disturbance, inhibition of root elongation, and finally, cell death (Bais et al., 2003; Weir et al., 2004).

Amur honeysuckle (*Lonicera maackii*), another invasive species with strong allelopathic potential, is known to produce 13 phenolic compounds in its leaves, which contribute to its success (Cipollini et al., 2008). These metabolites include phytotoxic luteolin and apigenin derivatives, which can inhibit seed germination and seedling growth of native species, highlighting their role in the invasive success of honeysuckle (Barta et al., 2023). The resistance of some species to the impacts of honeysuckle allelochemicals is linked to the elevated synthesis of gibberellic acid.

Most allelochemicals exert their effects by disrupting hormonal signaling, inhibiting cell division, impairing membrane integrity, and inducing oxidative stress in target species (Inderjit and Duke, 2003). These same mechanisms also make allelochemicals attractive candidates for integrated pest management and sustainable agriculture strategies aimed at reducing the ecological footprint of chemical inputs. Some allelochemicals have been shown to simultaneously improve both abiotic and biotic stress tolerance when used at low concentrations, suggesting a potential dual opportunity for managing salinity and protecting crops (Kaur et al., 2017; Kong et al., 2019).

The role of allelopathy in mitigating salinity stress was first proposed by Mahmood et al. (1989), who demonstrated that allelopathy can positively influence plant growth and distribution in saline environments. Since then, other studies have revealed multiple beneficial impacts of several classes of allelochemicals in ameliorating the harmful effects of high salinity on various plant species. The antioxidant potential of phenolic compounds has been linked to tolerance against heat, drought, and salinity stress (Farooq et al., 2010). Under salinity stress, phenolic acids—as ferulic and *p*‐coumaric acids—stabilize membranes and scavenge ROS (Cheng et al., 2024). Exogenous application of ferulic acid—naturally synthesized by many crops—enhanced salt stress tolerance in cucumber (*Cucumis sativus*; Li et al., 2013) and Chinese cabbage (*Brassica rapa* subsp. *pekinensis*; Linić et al., 2021) by improving antioxidant enzymatic activities and reducing membrane lipid peroxidation. Exogenous *p*‐coumaric acid similarly reduces the adverse effects of salinity stress in wheat (*Triticum aestivum*), improving germination and seedling development (Saleh and Madany, 2015). Applied in controlled experiments to glycophytes, catechins have been found to improve salt tolerance by controlling ROS generation and stabilizing photosynthetic efficiency (Bais et al., 2003; Li et al., 2010). Benzoxazinoids, such as DIMBOA, which are primarily produced by maize and grass species, have been implicated in enhancing root architecture and promoting resilience under saline and osmotic stress conditions (Niemeyer, 2009; Kong et al., 2019).

Due to their robust control over the health and establishment of neighboring species, allelopathic metabolites have also been used in agriculture (Zeng, 2014) to promote the development and selection of crops that require fewer chemical residues, thereby easing wastewater treatment needs and promoting recycling (Macias et al., 2003). In addition, allelochemicals are also promising alternatives to synthetic herbicides, although their efficiency and specificity may be limited (Bhadoria, 2011) due to their chemical instability and rapid degradation in the soil, their potential metabolism by rhizobacterial communities, and difficulties associated with their large‐scale production and formulation, which require advances in stabilizing the compounds and optimizing their delivery (Duke et al., 2000; Ain et al., 2023).

In addition to the beneficial effects of allelochemicals, their potential negative impacts also need to be considered when designing novel applications. As mentioned earlier, these chemical “weapons” inhibit the growth of native plants (Bais et al., 2003; Hierro and Callaway, 2003; Chen et al., 2017), facilitating the success of the invasive producers such as diffuse and spotted knapweed (Bais et al., 2003; Hierro and Callaway, 2003), mile‐a‐minute (*Mikania micrantha*; Shao et al., 2005; Wu et al., 2009), and Canada goldenrod (*Solidago canadensis*; Abhilasha et al., 2008; Yuan et al., 2013). To protect biodiversity, precise management strategies are needed to prevent the spread of invasive species. Advancing their targeted use in sustainable agriculture, especially in applications aiming to improve crop resilience under salinity stress, depends on understanding the unique chemical nature and ecological roles of allelochemicals.

The above examples suggest that conventional allelochemicals, often investigated for their herbicidal or defensive properties, can also be strategically utilized to enhance crop resilience by priming physiological and biochemical defense pathways. Harnessing these natural metabolites represents a promising avenue for developing sustainable and low‐input agricultural practices tailored to saline environments.

### Allelochemical‐like signaling molecules

Other endogenous molecules with primarily signaling functions in planta can also cause allelochemical‐like effects in specific conditions. In addition to these endogenous chemicals, exogenous allelochemical‐like molecules can also significantly affect and mitigate the deleterious effects of environmental stresses, including salt stress by enhancing the morphophysiological and biochemical adaptability of plants (Lutts et al, 1996; Ali, 2000; Wang et al., 2007; Sánchez‐Mareiras et al., 2009; Farooq et al., 2011b; Sarwar et al., 2019; Ullah et al., 2020). These nuanced, overarching roles of these endogenous and exogenous biomolecules in signaling highlight and support their potential as natural, plant‐derived agents to enhance resistance against stressors, including salt (Kong et al., 2019; Cheng et al., 2024).

Among these allelochemical‐like signaling molecules, phytohormones and polyamines are the most notable, with positive effects on salt stress tolerance in plants. The phytohormones salicylic acid (SA), jasmonic acid (JA), and ethylene play significant roles in regulating plant growth, development, and responses to stress (Abouelsaad and Renault, 2018; Kong et al., 2019; Wang et al., 2021; Li et al., 2022; Cheng et al., 2024). These hormones and their derivatives, methyl salicylate (MeSA) and methyl jasmonate (MeJA), primarily function in plant defense and stress signaling in planta; however, when released into the environment, they can impact neighboring plants (Shulaev et al., 1997; Park et al., 2007; Cheng et al., 2024).

The phenolic SA is synthesized via the shikimate pathway and has a fundamental role in local and systemic acquired resistance and in modulating antioxidant enzymatic activities during salt stress. Although SA is an endogenous signal, it can also be secreted into the rhizosphere or emitted into the air as methyl salicylate (MeSA) (Park et al., 2007; Vlot et al., 2009). The two forms serve distinct but complementary functions. Salicylic acid triggers intracellular defense and heightened antioxidant responses; MeSA serves as a volatile signal in long‐distance plant–plant communication and induces systemic tolerance in neighboring plants (Shulaev et al., 1997; Park et al., 2007). The exogenous application of SA alleviates salt‐induced oxidative stress by upregulating antioxidant enzymes, such as superoxide dismutase (SOD), catalase (CAT), and ascorbate peroxidase (APX), and by stabilizing photosynthesis (Khan et al., 2015; Cheng et al., 2024). In contrast, MeJA, the volatile derivative of JA, modulates stress tolerance by controlling defense‐related gene expression, promotes the accumulation of osmoprotectants (e.g., proline and soluble sugars), and upregulates the antioxidant system (Wasternack and Song, 2017). Although SA, JA, and their derivatives enhance salt tolerance, their primary modes of action differ. SA controls redox homeostasis and systemic resistance, whereas JA regulates stress hormone signaling cascades and metabolic adjustments, stimulating stress resistance.

Salicylic acid has been shown to improve salt tolerance in wheat, rice, and tomato (*Solanum lycopersicum*) (Jini and Joseph, 2017; Abdi et al., 2022; Fairoj et al., 2022; Pai and Sharma, 2022). Table 1 includes multiple examples of studies that show exogenous SA enhances growth, antioxidative capacity, and photosynthetic activity in a variety of plant species in saline conditions (Husen et al., 2018; Pai and Sharma, 2022; Salih et al., 2022; Kwon et al., 2023; Talaat et al., 2023). For example, pretreatment with exogenous SA reduces physiological and biochemical damage in salt‐stressed Ethiopian mustard (*Brassica carinata*) and argel (*Solenostemma argel*) and improves growth (Husen et al., 2018; Salih et al., 2022).

**Table 1:** Representative studies on the exogenous application of key allelochemicals and other molecules with allelopathic effects, illustrating their influence on plant responses to salinity stress.

Allelochemical (highest concentration)	Application mode	Plants	NaCl (highest concentration, mM)	Ameliorative effect	Reference
BOA (1 mM)	Soil	*Lactuca sativa*	60	RWC *F*_v_/*F*_m_	Sanchez‐Mareiras et al. (2009)
Cinnamic acid (50 µmol/L)	Vermiculite	*Cucumis sativa*	200	RWC Chl content	Wang et al. (2007)
Putrescine (0.01 mM)	Seed treatment	*Atropa belladonna*	50	Morphological features (germination) RWC	Ali (2000)
Putrescine (0.1 mM)	Foliar spray	*Oryza sativa*	50	FW	Lutts et al. (1996); Gil and Tuteja (2010)
*Helianthus annuus* plant extract (20%)	Seed treatment	*Oryza sativa*	50	Morphological features (germination, seedling growth, leaves number, FW, and DW)	Farooq et al. (2011b)
Triacontanol (0.80 mg/L)	Foliar spray	*Cucumis sativus*	50	Morphological features (shoot and root length, FW, and DW) Gas exchange, photosynthetic activity, *g*_s_ Membrane permeability maintenance	Sarwar et al. (2019)
Chitosan (150 mg/L)	Foliar spray	*Lycopersicon esculentum*	150	Morphological features (plant height, leaf area, stem diameter, fruit number and firmness, plant yield) Chl content Total soluble solids	Ullah et al. (2020)
*Sorghum bicolor* plant extract (2%) Vermicompost (10%)	Foliar spray Soil	*Zea mays*	120	Morphological features (FW and DW) Chl content Antioxidant system Leaf and root K^+^/Na^+^ ratio, and K^+^ contents	Alamer et al. (2022)
*Acacia dealbata* bark extract (450 ppm)	Foliar spray	*Allium cepa*	120 mm/L = EC 6.53 + ‐0.052 mS/cm	Morphological features (plant height, leaf expansion, root length, total biomass) Sugar and protein content	Lorenzo et al. (2019)
SA (0.5 mM)	Foliar spray	*Brassica carinata*	150	Morphological features (root, stem, leaves growth, biomass) Chl, carotenoid content *F*_v_/*F*_m_, *g*_s_, Pn, E, WUE Antioxidant system	Husen et al. (2018)
SA (0.5 mM)	Foliar spray	*Oryza sativa*	120	Morphological features (root, shoot length, biomass) *F*_v_/*F*_m_, PSII RWC Proline Antioxidant system (SOD)	Pai and Sharma (2022)
SA (0.362 mM)	Seed treatment	*Solenostemma argel*	100	Morphological features (shoot DW) Soluble protein Antioxidant system (SOD, POD)	Salih et al. (2022)
SA (0.25 mM)	Foliar (leaf disk) application	*Hypericum perforatum*	150	Morphological features (root length, plant height, stem diameter, FW) *F*_v_/*F*_m_, PSII, PSI Antioxidant system (SOD, DPPH, polyphenol, flavonoid)	Kwon et al. (2023)
SA (100 mg/L) + Spermine (30 mg/L)	Foliar spray	*Triticum aestivum*	120	Morphological features (leaf area, root, shoot growth, grain number, and yield) Nutrient uptake Chl content Osmolytes, leaf water content Antioxidant system	Talaat et al. (2023)
JA (0.1 uM)	Foliar spray	*Triticum aestivum*	150	Gene and transcriptional regulation Hormone biosynthesis Antioxidant system	Zhu et al. (2022)
IAA (200 mg/l)	Seed treatment	*Triticum aestivum*	15 S/m	Germination percentage Free salicylic acid accumulation	Iqbal and Ashraf (2007)
IAA (2 mM)	Foliar spray	*Zea mays*	100	Kernel yield	Kaya et al. (2013)
ABA (10^‐5 ^M)	Seed treatment	*Triticum aestivum*	100	Grain yield Grains/spike number	Gurmani et al. (2007)
BA (10^‐5 ^M)	Seed treatment	*Triticum aestivum*	100	Grain yield Grains/spike number	Gurmani et al. (2007)
Sorghum plant extract (5%) + BAP (5 mg/L)	Seed treatment	*Triticum aestivum*	100	Morphological features (plant height, leaf expansion)	Bajwa et al. (2018)
Spd (0.25 mM)	Foliar spray	*Brassica napus*	150	Chl content Proline PSII activity Rubisco activity Antioxidant system (APX, CAT, SOD, GR, DHAR) Polyamine pathway (PAO, DAO transcription) Calvin cycle enzyme‐related genes	ElSayed et al. (2022)

*Note*: ABA, abscisic acid; APX, ascorbate peroxidase; BA, benzyladenine; BAP, benzylaminopurine; BOA, benzoxazolin‐2(3H)‐one; CAT, catalase; Chl, chlorophyll; DAO, diamine oxidase; DHAR, dehydroascorbate reductase; DPPH, 2,2‐diphenyl‐1‐picrylhydrazyl; DW, dry mass; E: transpiration rate; *F*_v_/*F*_m_, photochemical yield of PSII; FW, fresh mass; GR, glutathione reductase; *g*_s_, stomatal conductance; IAA, indole‐3‐acetic acid; JA, jasmonic acid; PAO, polyamine oxidase; Pn, photosynthetic rate; POD, peroxidase; PSI, photosystem I; PSII, photosystem II; RWC, relative water content; SA, salicylic acid; SOD, superoxide dismutase; Spd, spermidine; WUE, water‐use efficiency.

Zhu et al. (2022) found that JA application ameliorates salt tolerance in wheat by regulating endogenous hormone balance and activating antioxidant defenses.

Indole‐3‐acetic acid (IAA) also alleviates salt stress‐induced damage in crops. After IAA treatment, the growth of wheat, maize, and barley (*Hordeum vulgare*) remains steady in saline conditions (Gurmani et al., 2007; Iqbal and Ashraf, 2007; Kaya et al., 2013; Quamruzzaman et al., 2021). In addition, abscisic acid (ABA), cytokinins, and auxins, through their key regulatory control over stomatal closure, osmotic regulation, and hormonal signaling, enhance water‐use efficiency (WUE) in plants during salt stress (Yu et al., 2020; Bharath et al., 2021; Balasubramaniam et al., 2023). Ethylene and ABA are both signaling molecules in plant stress management, regulating gene expression and helping to maintain cellular homeostasis in saline environments (Verma et al., 2021).

These findings underscore the fundamental contributions of plant hormones in modulating plant salt stress responses as endogenous signals and exogenous agents with allelochemical effects. Altogether, these findings suggest that natural, plant‐derived biomolecules, including signal molecules such as SA, JA, and other phytohormones, can be strategically harnessed to enhance abiotic stress tolerance in crops and advance sustainable agriculture.

Polyamines primarily act as endogenous regulators of plant growth, cellular metabolism, and stress responses. However, recent evidence suggests that they may also indirectly influence rhizosphere interactions by modulating plant exudates and oxidative balance (Alcázar et al., 2010; Yang et al., 2024). Therefore, polyamines also function as allelochemical‐like molecules, with beneficial effects on stress responses, including enhanced tolerance to salinity. Polyamines, including putrescine, spermidine, and spermine, are ubiquitous molecules that accumulate in plants under abiotic stress conditions (Alcázar et al., 2010). Plants treated with polyamines showed enhanced photosynthetic efficiency and diminished oxidative damage under salt stress (Chattopadhyay et al., 2002; ElSayed et al., 2022; Talaat et al., 2023). Polyamines play roles in stabilizing membranes, scavenging ROS, and modulating homeostasis of ions under stress conditions (Alcázar et al., 2010). Spermine and spermidine mitigate damage caused by saline environments in wheat, rice, and rapeseed (*Brassica napus*). The exogenous application of spermidine enhances the activity of antioxidant enzymes (e.g., SOD, CAT, and POD) in salt‐stressed wheat seedlings, improves seedling osmotic adjustment, and enhances their relative water content (ElSayed et al., 2018; Raziq et al., 2022). Similarly, pretreatment with putrescine increased K^+^/Na^+^ ratios and reduced electrolyte leakage in salt‐stressed rice (Ndayiragije and Lutts, 2007) and tomato (Najafi et al., 2025), thereby protecting cellular integrity. The ability of polyamines to stabilize membranes, detoxify ROS, and regulate stress‐responsive metabolic pathways highlights their potential as effective biological agents for improving crop salt tolerance. Research is needed to optimize treatment concentrations, delivery strategies, and timing of applications for maximal effects in specific crops in saline environments.

Plant extracts and tissues that degrade in the soil often contain or release a “cocktail” of allelopathic agents, which collectively benefit the recipient plants' salt stress resistance. These extracts or tissues may contain a mixture of different conventional allelochemicals, phytohormones, and polyamines, which synergistically trigger stress tolerance through their impacts on antioxidant enzyme activities, membrane stability, and phytohormone balance (Bonanomi et al., 2006; Cheng et al., 2024). A sunflower (*Helianthus annuus*) extract improves rice morphological traits during salinity stress and was attributed to the phenolic compounds, including chlorogenic acid, caffeic acid, and flavonoids, which enhance antioxidant defense and reduced oxidative damage in the rice plants (Farooq et al., 2011b). Similarly, the application of sorghum (*Sorghum bicolor*) extracts reduced salinity‐induced damage in wheat and maize, owing to the sorgoleone content in the extract. Sorgoleone is a potent allelopathic hydrophobic benzoquinone that inhibits lipid peroxidation and stabilizes cell membranes under stress (Bajwa et al., 2018; Alamer et al., 2022). Silver wattle (*Acacia dealbata*) extracts counteract the adverse effects of salinity in onion (*Allium cepa*) through an effect linked to the flavonoids quercetin and kaempferol derivatives in the extract. These allelochemicals scavenge ROS and modulate osmotic balance (Lorenzo et al., 2019) (Table 1). Recent studies by Barta et al. (unpublished manuscript) revealed that velvet bean (*Mucuna pruriens*) seed amendments in the soil enhance the growth and photosynthetic energy utilization capacity of tomato, Russian red kale (*Brassica napus* var. *pabularia*), and big bluestem (*Andropogon gerardii*). These amendments also induce salinity tolerance in tomato plants, which were irrigated with saline water containing up to 400 mM NaCl. Barta et al. (unpublished manuscript) attribute these salinity tolerance effects to the action of allelochemicals released by the velvet bean seed, which reduces sodium uptake in tomato roots.

Ultimately, the application of plant‐derived extracts rich in bioactive secondary metabolites, combined with soil enrichment using plant tissue amendments, offers a natural and multifaceted approach to mitigating salt stress, thereby supporting sustainable crop production under increasingly challenging soil conditions.

Recent evidence also suggests that plant‐growth‐promoting rhizobacteria (PGPR) also synthesize a large variety of molecules with allelopathic functions and significantly influence rhizosphere ecology, promoting plant growth and stress tolerance through mechanisms that involve the production and release of allelochemicals and allelochemical‐like molecules. Different PGPR strains of the genera *Enterobacter*, *Bacillus*, *Azospirillum*, and *Pseudomonas* were noted to produce various molecules with allelopathic effects. These include IAA, siderophores, exopolysaccharides (EPS), 1‐aminocyclopropane‐1‐carboxylate deaminase (ACCD), and volatile organic compounds (VOCs) such as 2,3‐butanediol and acetoin. These molecules were suggested to alleviate salt‐induced damage in maize, wheat, soybean, barley, tomato, and potato (*Solanum tuberosum*). Recipient plant species benefit from PGPR‐produced biomolecules such as IAA, siderophores, and ACCD because these molecules enhance their salt tolerance through their impact on processes that control osmotic adjustments, the antioxidant network, and nutrient uptake and mobilization (Mahmoud et al., 2020; Hamid et al., 2021; Giannelli et al., 2023; Kaushal et al., 2023; Kumawat et al., 2023). The allelochemicals and allelochemical‐like biomolecules produced by PGPRs have been shown to enhance plant growth, photosynthetic efficiency, and antioxidant activity under saline conditions. Salt tolerance is further strengthened by the ability of PGPR to improve nutrient uptake and soil structure through the production of exopolysaccharides and biofilms. Khan et al. (2020) reported that inoculating salt‐stressed wheat with a consortium of PGPR significantly alleviated salt‐induced damage by promoting root growth, enhancing antioxidant defense systems, and improving nutrient acquisition. These findings suggest promising applications for PGPR‐derived allelochemicals and biomolecules with allelochemical‐like effects in enhancing crop resilience and productivity in saline environments, offering sustainable biological alternatives to traditional chemical‐based approaches.

**Figure 4:**
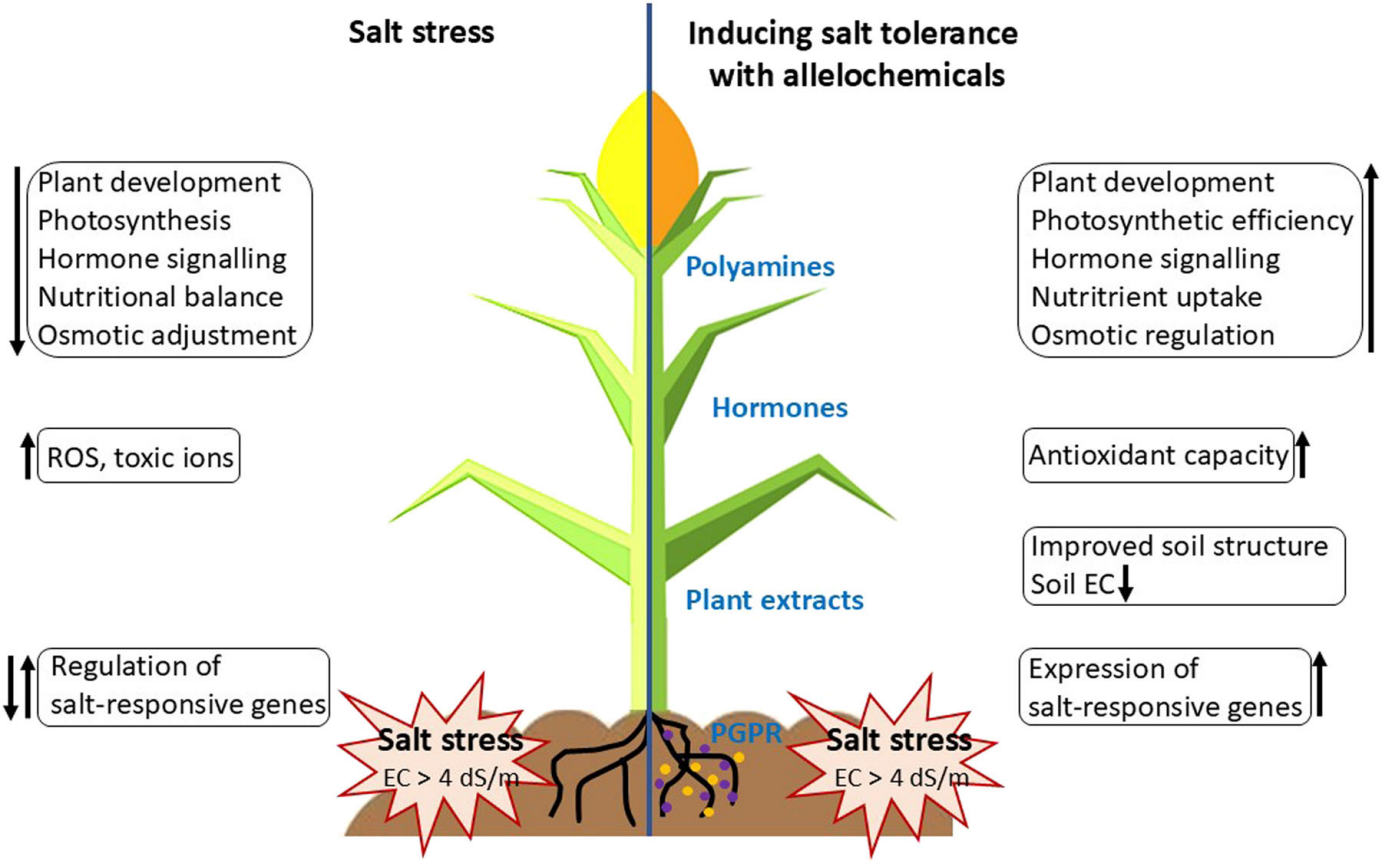
Modeled impacts of salt stress on plants and the mitigating effects of allelochemicals and allelochemical‐like signaling molecules. Arrows indicate the direction of the regulation. dS/m: deciSiemens per meter; EC: electrical conductivity; PGPR: plant‐growth‐promoting rhizobacteria; ROS: reactive oxygen species.

Ultimately, the use of allelochemicals and other molecules with allelopathic effects represents a promising strategy for enhancing salt stress tolerance and improving crop yield in salt‐affected soils. Table 1 presents representative studies on the exogenous application of key allelochemicals and other molecules with allelopathic effects, illustrating their influence on plant responses to salinity stress, and the graphical model in Figure 4 summarizes the impacts of salt stress on plants and the mitigating effects of allelochemicals and allelochemical‐like signaling molecules.

Here, we propose that halophyte‐produced allelochemicals be exploited in agricultural applications, which could enable the co‐cultivation of salt‐sensitive crops with halophytes. Such an approach may enhance crop salinity tolerance and adaptation through biochemical interactions with naturally salt‐tolerant neighbors. For example, priming and inducing salt tolerance in non‐tolerant species in this manner could open novel avenues for food crop production and support the reclaiming of currently salinized soils. Conventional allelochemicals, phytohormones, polyamines, plant extracts, tissue derivatives, and PGPR‐produced molecules can regulate key physiological and biochemical processes, including the antioxidant defense network, ion homeostasis, and nutrient acquisition, ultimately improving crop productivity under saline conditions. Thus, the use of allelochemicals could offer a sustainable solution to the global challenge of soil salinization and food security. Future research is expected to determine optimal application methods tailored to specific crops, soil types, and environmental conditions. A careful assessment of potential risks and unintended effects on nontarget organisms and ecosystems is fundamental to ensure the safe and sustainable use of allelochemicals in agriculture.

## FUTURE DIRECTIONS

One major approach to solving food shortages as soil salinization, population growth, and food demands increase (FAO, 2021; IPCC, 2023) is the development of salt‐tolerant crops in salinized regions (Rozema and Flowers, 2008; Shabala, 2013). Another promising and complementary strategy is the harnessing of allelopathic plant–plant interactions, in which plants produce and release chemical compounds that affect the growth, development, and survival of neighboring species (Weston and Duke, 2003; Kong et al., 2019). Recent studies have demonstrated that the use of allelochemicals can be a creative and environmentally friendly approach to mitigating salt stress, particularly in areas affected by extensive soil salinization (Abbas et al., 2021; Cheng et al., 2024). Future studies should prioritize the identification and mechanistic characterization of new allelochemicals that enhance plant adaptability, stress tolerance, and yield in saline conditions. The advancement of practical applications depends on foundational knowledge at cellular, physiological, biochemical, and molecular levels (Kong et al., 2019; Cheng et al., 2024). Along with these efforts, environmental risk analyses are needed to assess allelochemical treatments for any adverse effects on rhizosphere microbial communities, nontarget organisms, and ecosystem health (Duke et al., 2000; Macías et al., 2003).

Several approaches can be suggested for the direct use of allelochemicals derived from halophytes. (1) Halophytes can be intercropped with sensitive crops so that allelochemicals are released into the shared rhizosphere, potentially improving the salinity tolerance of the sensitive species (Simpson et al., 2018; Bazihizina et al., 2024). Comparing such halophyte–glycophyte mixtures with glycophyte and halophyte monocultures across salinity gradients could be a feasible experimental design and support future agricultural applications. (2) Pretreating crop seeds with purified allelochemicals or halophyte extracts can induce physiological and biochemical priming, thereby improving seedling vigor and early resilience to salt stress (Ashraf and Foolad, 2005; Farooq et al., 2006). Germination rates, early growth, and oxidative stress markers in primed seeds need to be compared to nonprimed seeds in controlled environment and field trials. (3) Short‐cycle halophytes could be used as cover crops for the gradual, continuous release beneficial allelochemicals into the soil, and consequently, improve soil structure, lower salt accumulation in the root zone, and enhance the resilience of crops planted in the subsequent rotation (Hasanuzzaman et al., 2014). (4) Bioformulas and soil amendments can be developed for targeted delivery of allelochemicals encapsulated in controlled‐release matrices (e.g., polymer beads, nanoparticles) to minimize environmental losses and leaching while extending their recipient‐targeted activity in the soil (Vinale et al., 2008; Kumar et al., 2022; Khan et al., 2023). (5) Screening and breeding for allelochemical traits is needed for breeding programs to produce crops better suited to saline soils and identify crop varieties with natural abilities to produce or react favorably to allelochemicals (Olofsdotter et al., 2002; Ain et al., 2023). (6) Halophytes that recruit stress‐resilient microbial communities should be harnessed to enhance allelochemical potential and improve crop growth and performance in saline environments (Etesami and Beattie, 2018; Mahmoud et al., 2020).

Technologically, maximizing field efficacy and reducing volatilization, leaching, or degradation losses will depend on the development of effective delivery systems, including encapsulation technologies or seed coatings with allelochemicals. Investigating synergistic combinations of allelochemicals with biofertilizers, soil conditioners, or organic additions might result in integrated, multifaceted approaches for sustainable salinity management. Furthermore, the complete understanding of how allelochemicals affect rhizosphere microbial communities is fundamental. Under saline stress, allelochemicals or similar molecules may not only affect plant physiology but also have beneficial impacts on microbial consortia, thereby improving plant–microbe symbioses (Vurukonda et al., 2016; Etesami and Beattie, 2018).

Laboratory results need to be validated with extensive field trials across different salinity‐prone regions, soil types, and crop systems. These trials should incorporate evaluations of microbial diversity and soil factors and crop productivity for long‐term sustainability. Advancements depend on cooperative, multidisciplinary research that incorporates plant physiology, biochemistry, molecular biology, chemistry, soil science, and agricultural engineering. Priority mechanistic research is expected to translate laboratory discoveries into field innovations, ultimately advancing the adoption of allelochemical‐based approaches for enhancing food security and environmental resilience through partnerships among scientists, legislators, and agricultural stakeholders. In addition, successful outreach and farmer education initiatives must accompany scientific developments to ensure the widespread acceptance, appropriate application, and long‐term sustainability of these new biological solutions.

We suggest that the allelopathic potential of halophytes and their strategies for resisting salt stress present a promising opportunity for mitigating the negative impacts of salinity stress in sensitive food crops. Allelochemicals could transform salinity management techniques if more research and technological developments are pursued, significantly contributing to a more resilient and sustainable world agricultural system.

## AUTHOR CONTRIBUTIONS

G.S. and C.B.: conceptualization; analysis; writing and editing all drafts.

## CONFLICT OF INTEREST STATEMENT

The authors declare that they have no conflicts of interest.
